# Mouth Hygiene*Read before the Southern California Dental Association, June 21, 1921.

**Published:** 1921-09

**Authors:** Guy S. Millberry


### MOUTH HYGIENE*

BY GUY S. MILLBERRY, D.D.S.,

A verse by James Whitcomb Riley recently published seems a fitting introduction to my subject:

> "Take the best man ever wuz,
> Nigh 'bout dead and Heaven in sight,
> He don't want no Infinite,
> What he wants is—Health and the Doctor,
> And he's right."

Health and the doctor, every-day terms; our interests are involved in both.

Dr. Alfred C. Fones of Bridgeport, Connecticut, has graphically portrayed the health situation in a diagrammatic way, which points the way quite clearly toward one phase of our professional activity, viz: mouth hygiene and preventive dentistry.

*Read before the Southern California Dental Association, June 21, 1921.

RECENT DEVELOPMENTS.

It is hardly worth while to review the history of the mouth hygiene movement. It is familiar to most of you. Recent additions to our records show Pennsylvania, Arkansas, and California in the group of states[1], making fifteen, which have already passed laws licensing the dental hygienists, with Ohio and Illinois having statutes pending gubernatorial action.

Another notable movement in this direction is the gift of Mrs. George R. Carter, of Honolulu, of the sum of $1,000,000 for the erection and maintenance of a dental infirmary and training school for dental hygienists at Honolulu. This act has been publicly endorsed by the passage of a law licensing dental hygienists there, and the appropriation of $20,000 annually by the Hawaiian Legislature for the compensation of these women who are to serve in the public schools. The local benefits to be derived from this plan are certain to be commensurate with the private and public benefactions, for the interest of the people is well aroused.

With as large a foreign population, especially Japanese, as is found in these islands, this notable philanthropic effort will undoubtedly carry American dentistry into the Orient more rapidly and thoroughly than any movement yet inaugurated; indeed, it is expected that such a great good may evolve out of it. Dr. and Mrs. Fones have recently visited the islands at Mr. and Mrs. Carter's request in order to convey to those interested, the plans and results of the work accomplished in this country.

PROFESSIONAL ACTIVITY

The professional activity in the field of mouth hygiene has been sporadic among both individuals and associations.

---

[1] States where dental hygiene is legalized: 1, Connecticut, 1915-1917; 2, New York, 1916; 3, Massachusetts, 1917; 4, Maine, 1917; 5, Iowa, 1917; 6, Oklahoma, 1919; 7, Colorado, 1919; 8, Tennessee, 1919; 9, Alabama, 1919; 10, Minnesota, 1919; 11, Michigan, 1919; 12, New Hampshire, 1919.

Here and there an individual becomes imbued with a desire to do some good work, or inspired by the results of the work of others, and an amount of energy is loosened in a community or state calculated to accomplish wonders, or one with a keen understanding of the value of this work quietly exercises his influence with boards of supervisors, or other public officials, or arouse the interest of civic bodies, such as women's clubs, the Rotarians, Kiwanis, Lion's Clubs, and the like until the project becomes known and perhaps supported as a public necessity.

The most democratic and far-reaching plan yet proposed that has come under my observation is that proposed by Dr. C. H. Henderson of Martinez, Contra Costa County, California. His vital interest in the problem for the past six years has brought him the chairmanship of the oral hygiene committee of his county society, which with the aid of the Women's Federation of the county is proposing to add a tax of five cents on the hundred dollars for mouth hygiene work throughout the county. It is estimated that $50,000 annually will be raised by this method, which will provide a service more complete and efficient than any yet promulgated. The proposal is worthy of our consideration.

### ASSOCIATIONAL ACTIVITY

Associational activity usually takes the form of a committee appointment with surveys—ways and means proposals, publicity, equipment purchases, and some form of service. The procedure is more or less stereotyped, following established methods adopted in other places. The effect is good as far as it can be carried out, but owing to the limitations of service provided by one or more pairs of hands, the results are not wholly satisfactory. The constantly increasing demand for dental service and the lessening number of licentiates at present available is another factor contributing to the failure of this procedure as at present formulated.

A notable achievement was accomplished by the Chicago Dental Society at its last annual meeting, January 27-28-29, 1921. Under a committee headed by Dr. Arthur D. Black, the principal institutional exponents of mouth hygiene in America presented their methods and policies in the form of continuous clinics and discussions, passing in review before seated audiences.

Discussions and demonstrations were presented by Dr. Alfred C. Fones, Mrs. Hubert Hart, Miss A. Cunningham, and Miss Cortwright, on the Bridgeport plan including tooth-brush drills, school dental hygiene, and private office practice.

The Forsyth Dental Infirmary was represented by Dr. Harold DeWitt Cross, Dr. Paul K. Titus, and Miss Evelyn Schmidt, who similarly presented the plan followed at the great institution in Boston.

Dr. Eda B. Schlenker presented the work as carried on at Rochester in their dental dispensary, touching upon the orthodontic need and procedure as well as the school and clinic dental service.

Dr. Anna V. Hughes, director of the school for dental hygienists of Columbia University, represented that institution with Miss Violette Durrant, who presented their methods.

Chicago was represented by its school dental staff and nurses who demonstrated the work to be done and being done by the presentation of "Before and After Cases." They also supplied the children for the other clinics.

Drs. C. M. Kennedy, W. J. Cameron and W. J. Charters of Des Moines, Iowa, showed the work carried on in their state, which had been presented at the New Orleans meeting of the National Dental Association, as a plan to teach the dental profession how to teach their clientele the proper methods of practicing mouth hygiene.

At no time before this had all the great influences in this forward looking movement on the part of our colleagues been gathered together in one program. The afternoon was given over to a repetition of the program for the benefit of the teachers and public school nurses, the schools being closed for that half day, and a large public meeting was held in the Auditorium Theatre at which more than 2,000 people were present, the speakers being Dr. William A. Evans, health editor of the Chicago *Tribune,* and Dr. Alfred C. Fones. The day's events closed with a testimonial banquet to Thomas Alexander Forsyth.

PUBLICITY

Publicity, the next important phase of this work, is generously given by the press, and is of a higher educational and professional tone than in the past. A news writer recently stated that it is no longer necessary to convince the reading public of the importance of the care of the mouth and teeth; every sensible person believes it.

Our publicity problem is not confined, however, to the newspaper reading public. The message must be gotten over to the youngest group of readers we have, the kindergarten and first grade children, hence other forms of publicity than news, must be made use of.

The California State Dental Association through its educational committee has been fortunate in arousing the interest of the leading social welfare and health organizations in the state in this work so that now they are spreading the gospel of ''clean mouths, teeth and healthy gums,''[2] all over the state. To illustrate: a dental hygienist accompanied the officers of the Bureau of Child Hygiene of the State Board of Health into the northern countries in June, 1920. Sixteen children presented themselves for examination and prophylaxis at one of the conferences in the small village of Williams. This year 153 children were brought for a similar survey and advice. There is no dental service available within 30 miles.

---

2 Dr. Alfred C. Fones' slogan.

The development of any form of publicity should be influenced largely by our state associations through committees co-operating with the organization which will probably carry on this work in the future, namely, the State Board of Health.

### LEGISLATION

This brings me to the subject of legislation. I believe that we will nearly all agree that the success of the mouth hygiene movement depends upon three things: (1) The prophylactic service for the children with the personal instruction accompanying it; (2) the education of the public in this great social work, and (3) the guidance of the effort by the dental profession.

To these ends it became necessary to provide for the first thing by the enactment of legislature. By repeatedly bringing the matter to the attention of the dental profession and to other interested organizations during the past two years, the fundamental principles of the dental hygienist's service was gradually unfolded. Throughout the state nothing but unqualified endorsements was received during that period.

The draft of the bill submitted to the Legislature two years ago was the basis of the bill passed this year, and with the exception of a few individuals in Alameda County, who strenuously opposed the bill after it had passed both houses of the legislature, no dissenting voice was heard.

Perhaps most of you have not read the law. The legislative committees made a conscientious effort to get it before you and if its language is not as explicit as you or I might like, nevertheless, it is capable of a reasonable interpretation by the state officials for it has been drawn to co-ordinate with our own dental practice act, and in accordance with the laws of other states. If it is not interpreted and enforced in a reasonable way, then it is the duty of the dental profession to inquire into the issue in order that our children may profit by the measure which is intended for their welfare.

Since the school is the most logical place to carry on this educational work, both by precept and practice, it was necessary to amend the school law. With the co-operation and assistance of the Superintendent of Public Instruction, Mr. Will C. Wood, this was accomplished and Boards of Education are now legally empowered to employ various health agents including dentists and dental hygienists to make surveys and care for the remediable defects of children in our schools at public expense.

The third measure which became a law provides for a division of dental hygiene in the State Board of Health. This division will be an information bureau to gather statistics from all parts of the state, nation, and the world and disseminate such to whomsoever may ask for it.

The dissemination will take the form of lectures, exhibits, posters, pamphlets, reports, outlines for surveys, record forms and any other development that time and funds will permit of.

It is proposed that less than one-third of the appropriation be spent on salaries, about one-sixth on traveling expenses, and the remainder on publicity, $7,500 per year being available.

With our co-operation, this division should become a valuable adjunct to the service of the state Board of Health in California.

May I suggest that the next step along these lines should be the appointment of a dentist on the State Board of Health. This has been discussed informally and editorially commented on in the PACIFIC DENTAL GAZETTE[3] recently and is deserving of associational consideration. We, in the north, are not concerned with the individuals, but only with the principles involved and we know that

---

[3] *Pacific Dental Gazette* for April, 1921.

men of good judgment and suitable qualifications are to be found in many localities.

SERVICE

In the furtherance of this plan of mouth hygiene, service is the foundation. This involves a study of the character of, training for, and utilization by all agents or agencies concerned. It devolves upon the dental profession to determine, by suggestion and criticism, the character of service to be rendered. That there will be transgression beyond the explicit terminology of the law may be expected in some cases, but those most interested believe that the training schools can inculcate a spirit of professional ethics in the minds of the trainees that will result in a product equally as, if not more ethical than the dentists themselves, hence there is little to fear. For this reason, I hope the Southern California school will soon inaugurate a course.

The training in most institutions, and I believe institutions should train the dental hygienist as well as the dentist, is specific insofar as dental anatomy, dental histology, and prophylaxis are concerned, and liberal insofar as other subjects, of which she should know something, are concerned.

Announcements of the course we propose to continue will soon be off the press for your information, if you are interested.

The utilization of the service in institutions is a thing to be encouraged, especially in schools. Your civic duty is thus clearly defined. Appointments should be made on the same basis as public school nurses and teachers on the year plan for the sake of permanency and continuity of service; the compensation problem is now being studied and a paper on that subject will be presented to the profession in the near future.

In private offices the question of status arises, whether it be professional with consideration as an associate, or industrial with consideration as an employee. The former seems to lead to the best results in the East, though each plan will develop largely as a result of the individuals concerned. It is hoped that those who choose to engage a dental hygienist will not lose sight of the opportunity to encourage the children to come regularly for service, since they have generally been uncared for until serious conditions arise.

### FOREIGN ACTIVITIES

Other countries have taken a very vital interest in this problem. The Canadians east of the lakes are very progressive and have accomplished much through their Canadian Oral Prophylactic Association, which later became a section of the Canadian Dental Association.

England is endeavoring to revolutionize dentistry for the betterment of her people in every way. New Zealand has passed a law with appropriations to provide limited classes of dental service for every child in the public schools. Australia has had its traveling school dental clinics for several years.

The movement is receiving favorable consideration in the Scandinavian countries. Stefanson, the explorer, states that since civilized foods have been introduced among the Icelanders, dental caries has developed rapidly where it did not exist before.

Japan is wide awake. Last year a quarter of a million hand-bills and flags were distributed in a single day, called Tootache Day, in Tokyo. The sixty-five dental associations and seven dental colleges are unitedly back of the movement.

### THE FUTURE

What is the forecast? Our perspective may be improved by looking back over a half century of progress. Prior to 1870, the physician had few public or social functions or responsibilities. They operated on the old plan that in order to cure disease it was necessary to treat humanity one at a time.

Today when the physician is confronted with a case of typhoid fever, he is not only concerned with the case itself, but is much more concerned with the source and spread of the disease in order that others may not become victims of it.

Where the medical profession "has in part failed to recognize its social responsibilities, other groups without thorough medical training are taking advantage of the opportunity."[4] Let us not become involved in a similar situation, but extend our social vision and expand our social consciousness so that humanity as well as our profession will benefit thereby.

This involves some study and leadership in social problems which becomes a part of the functions of our dental schools, and it should never be confused with social health insurance, which deals wholly with effects and not with causes.

Oliver Wendell Holmes, of the Harvard Medical School faculty, in a commencement address in 1872 on "The Claims of Dentistry," said: "but what would old age be without the aid of the optician and the dentist? The monument of civilization is a perpetual struggle between the arts to destruction on the one hand and of construction and conservation on the other. And if any of the arts of peace should have appeared entitled to the highest consideration of a civilized people, it would seem to be that which professes to relieve suffering and prolong life." Today "the social value of medical service is now equal to if not greater than the value of medical service to the individual."[5] Let us not fail to recognize our social responsibilities in order that we may fulfill the obligations expected of us.

---

4 Fred R. Green, *Journal of American Medical Association*, May 28, 1921.

5 Fred R. Green.

This problem opens up a field of dental activity that will demand the attention, interest, and undoubtedly the service of persons trained wholly or in part in dental science. The appointment of dentists and dental hygienists in public service such as the Bureau of Oral Hygiene, State Board of Health of Tennessee with A. S. Buckner, D.D.S., as director, and other similar positions in public and private institutions points the way toward which we must progress.

Our method of putting the message over to the children must be studied and evolved in a practical way. The recently developed plans with the health fairy, the clown, and other characters, are but instances of the possibilities before us.

As a result of the many years of experience in this field including six years in a thorough study of the school problem in Bridgeport, Dr. Fones proposed a law this year which was submitted to the Connecticut legislature. It was the direct result of the mouth hygiene movement and provided for the training of public school hygienists in the normal school of that state.

A course of training offered without tuition and including such subject matter as related directly to child hygiene was proposed. The graduates were obliged to serve the state for two years after graduation on salary in return for their instruction. This would place the teaching of hygiene on an equal footing with history, English, and mathematics in the normal schools of the state. With the interest in the health of our school children becoming more vital each year, especially in the parent-teacher organizations, a study of this proposed legislation during the next two years would not be inappropriate.

In conclusion, let us remind ourselves of certain economic conditions confronting all the people. Disease is the nation's greatest burden. It must be borne by those who are able to carry it. When the burden falls on the ignorant and poverty stricken, it becomes a public responsibility and others must bear it.

It is estimated that only one-third of our people are producers and two-thirds are dependents, and any added burdens from disease must therefore fall on the producers.

We are cognizant of the fact that most of our ills are the direct result of septic mouths, or are transmitted to the body by way of the mouth. We are also cognizant of the fact that clean mouths, sound teeth and healthy gums will inhibit if not eradicate most of these ills.—*Pacific Gazette,* July, 1921.

